# Site‐to‐Site Reproducibility and Spatial Resolution in MALDI–MSI of Peptides from Formalin‐Fixed Paraffin‐Embedded Samples

**DOI:** 10.1002/prca.201800029

**Published:** 2019-01-04

**Authors:** Alice Ly, Rémi Longuespée, Rita Casadonte, Petra Wandernoth, Kristina Schwamborn, Christine Bollwein, Christian Marsching, Katharina Kriegsmann, Carsten Hopf, Wilko Weichert, Jörg Kriegsmann, Peter Schirmacher, Mark Kriegsmann, Sören‐Oliver Deininger

**Affiliations:** ^1^ Bruker Daltonik GmbH Bremen Germany; ^2^ Institute of Pathology University Hospital Heidelberg Heidelberg Germany; ^3^ Proteopath GmbH Trier Germany; ^4^ Institute of Pathology Technical University of Munich Munich Germany; ^5^ Center for Biomedical Mass Spectrometry and Optical Spectroscopy (CeMOS) Mannheim University of Applied Sciences Mannheim Germany; ^6^ Department of Hematology Oncology and Rheumatology University Hospital Heidelberg Heidelberg Germany

**Keywords:** formalin‐fixed paraffin embedded tissue, MALDI, reproducibility, tissue typing, workflow

## Abstract

**Purpose:**

To facilitate the transition of MALDI–MS Imaging (MALDI–MSI) from basic science to clinical application, it is necessary to analyze formalin‐fixed paraffin‐embedded (FFPE) tissues. The aim is to improve in situ tryptic digestion for MALDI–MSI of FFPE samples and determine if similar results would be reproducible if obtained from different sites.

**Experimental Design:**

FFPE tissues (mouse intestine, human ovarian teratoma, tissue microarray of tumor entities sampled from three different sites) are prepared for MALDI–MSI. Samples are coated with trypsin using an automated sprayer then incubated using deliquescence to maintain a stable humid environment. After digestion, samples are sprayed with CHCA using the same spraying device and analyzed with a rapifleX MALDI Tissuetyper at 50 µm spatial resolution. Data are analyzed using flexImaging, SCiLS, and R.

**Results:**

Trypsin application and digestion are identified as sources of variation and loss of spatial resolution in the MALDI–MSI of FFPE samples. Using the described workflow, it is possible to discriminate discrete histological features in different tissues and enabled different sites to generate images of similar quality when assessed by spatial segmentation and PCA.

**Conclusions and Clinical Relevance:**

Spatial resolution and site‐to‐site reproducibility can be maintained by adhering to a standardized MALDI–MSI workflow.

## 1 Introduction

Preservation of clinical tissue samples is achieved by fixation in formalin and subsequent embedding in paraffin (formalin‐fixed paraffin‐embedded; FFPE). This process maintains excellent tissue morphology and allows indefinite sample storage at room temperature. As FFPE tissue specimens are routinely acquired during hospital care, it is imperative that tissue‐based diagnostic techniques are able to analyze such samples. While most commonly associated with histopathology and immunohistochemistry (IHC), FFPE tissues can be used for other analyses, such as next‐generation sequencing and MS/MS.^1^, ^2^ However, these methods often require large amounts of tissue and suffer from the loss of spatial information due to sample preparation procedures. In contrast, the spatial distribution of molecules is retained in MALDI–MS Imaging (MALDI–MSI). By combining histology and MS, MALDI–MSI has great potential in clinical pathology by allowing detection of multiple molecules in single intact tissue section and the creation of tissue or clinically specific molecular profiles, also known as tissue typing.

As intact proteins are not accessible for direct MALDI–MSI, tryptic peptides form the bulk of MALDI–MSI studies using FFPE samples. This has been particularly useful for investigating clinically relevant questions where typical histomorphological analysis may be ambiguous, e.g., classifying thyroid cancers,^3^, ^4^ identifying metastasis origin,^5^ and discriminating between benign and malignant skin lesions.^6^ MALDI–MSI has been used for retrospective studies, such as identifying proteins associated with lymph node metastases from primary endometrial cancer.^7^, ^8^ Another benefit is the possibility to perform classification on a single tissue section, thereby saving tissue material for subsequent analyses. For example, MALDI–MSI was able to differentiate between adenocarcinoma and squamous cell carcinoma (SqCC) of the lung,^9^ a condition where biopsy material is often limited, but required for additional predictive analyses.^10^

A number of obstacles still prevent the acceptance of MALDI–MSI as a clinical tool. First, the spatial resolution of peptide MALDI–MSI was inferior to traditional microscopy, with raster sizes for FFPE samples generally being between 50 and 100 µm.^3^, ^8^, ^9^, ^11^, ^12^, ^13^ Second, sample preparation protocols for peptide MALDI‐MSI of FFPE tissues require many steps that can potentially introduce variability.^12^, ^14^, ^15^ Added complications include: the variation in preanalytics with regard to the instrumentation, solvents, and paraffin used, and timing of each stage; individual laboratories tending to optimize their MALDI–MSI sample preparation and measurement protocols according to available instrumentation and research interest, and that few research projects source tissue samples from different sites. At present, there is one FFPE study in which reproducibility has been addressed at different sites.^16^ While one significant study demonstrated the ability to accurately diagnose diagnostically challenging atypical Spitzoid neoplasms FFPE specimens from samples collected from 22 sites around the world,^17^ these samples were measured at one centralized site. The importance of examining both inter‐site technical variations in terms of sample origin, preparation, and measurement therefore remains.

We present an integrated MALDI–MSI tissue typing workflow for tryptic peptides from FFPE tissues together with the first results from a multicenter study evaluating reproducibility. This protocol was developed with the aim of decreasing preparation time while maintaining spatial resolution and improving reproducibility. Using this system, specific *m/z* features are detected in discrete histological features in mouse intestine, human ovarian teratoma, and human squamous cell carcinoma of the lung. In the multicenter study, the delineation of histological features in the mouse intestine was demonstrated at five sites across two time points. These results demonstrate that strict adherence to a standard operating procedure can yield reproducible results between different sites, which is crucial to the acceptance of MALDI–MSI as a clinical technique.

## 2 Experimental Section

### 2.1 Tissue Collection

FFPE blocks of ileum and jejunum from adult C57Bl/6 mice were donated by the Institute of Pathology, Technical University Dresden. A human ovarian teratoma section was provided by ProteoPath GmbH (Trier, Germany). A tissue microarray (TMA) of six different human tumors selected for being examples of their type including mantle cell lymphoma, seminoma, squamous cell carcinoma of the lung, leiomyoma, breast cancer, and melanoma was constructed from tissues collected at the Institute of Pathology, University Heidelberg, Institute of Pathology at the Technical University of Munich, and ProteoPath. Patients provided informed, signed consent and all sites collected the tissues with approval from the respective ethics committee (reference no. from the biobank of the National Cancer Centre (NCT: 2097).

**Clinical Relevance**

MALDI–MS Imaging (MALDI–MSI) is a label‐free method that allows for the extraction of molecular information from tissue sections and can be used to create clinically relevant classification models, also known as tissue typing. Despite its potential application in pathology, broad acceptance of MALDI–MSI as a clinical technique has been hampered by a number of factors. Widespread clinical use of MALDI–MSI requires the ability to analyze formalin‐fixed paraffin‐embedded (FFPE) tissues, but additional sample preparation steps needed to prepare such sections for MALDI–MSI introduce variation and decrease spatial resolution compared to traditional light microscopy techniques. Additionally, data on reproducibility between measurements from different sites are largely lacking. We present an integrated MALDI–MSI workflow for proteomic analysis of FFPE samples resulting in improved spatial resolution as demonstrated on mouse intestine and human tissue samples. When applied in a multicenter study, five sites using the same protocol were able to delineate the same histological features in the mouse intestine. This demonstrates that MALDI–MSI of FFPE samples is reproducible when using a standardized workflow, which is a prerequisite for clinical applications.

All tissues were processed for paraffin embedding according to the respective automated protocols at each collection site. Tissue samples were fixed for 12–24 h in 10% neutral buffered formalin, dehydrated in graded ethanol, cleared in xylene, and embedded in molten paraffin. FFPE sections (3–5 µm) were mounted onto indium‐tin‐oxide (ITO) coated glass slides (Bruker Daltonik, Bremen, Germany) precoated with 1:1 poly‐l‐lysine (Sigma–Aldrich, Munich, Germany) and 0.1% IGEPAL CO‐630 (Sigma),^18^ dried overnight at 37 °C, and then stored at room temperature until MALDI–MSI analysis.

### 2.2 Sample Preparation

FFPE sections were annealed onto the ITO slides at 80 °C for 15 min, deparaffinized in xylene (2 × 5 min; Honeywell, Seelze, Germany), washed in isopropanol (Honeywell), and rehydrated in 100%, 96%, 70%, and 50% HPLC‐grade ethanol (5 min each; Honeywell). Heat‐induced antigen retrieval was conducted using de‐ionized water (ELGA Purelab Flex System; Veolia, Cell, Germany) in a decloaking chamber (Biocare Medical, Pacheco, CA, USA) or a Vitacuisine VS 4001 (Tefal; Dijon, France). Both antigen retrieval devices were used according to their established protocols at 110 or 98 °C respectively for 20 min. The sections were allowed to air‐dry before scanning at high‐resolution (TissueScout; Bruker).

Sequencing‐grade modified trypsin (Promega, Mannheim, Germany) was diluted to a final concentration of 25 µg mL^–1^ in 20 mm NH_4_HCO_3_ (Sigma–Aldrich, Munich, Germany) buffer solution containing 0.01% glycerol (Sigma), and deposited on the sample using a TM sprayer (HTX Technologies, Chapel Hill, NC, USA), in 16 layers using the parameters of 30 °C, 0.015 mL min^–1^ flow rate, and 750 mm min^–1^ velocity. Digestion chambers were prepared with saturated K_2_SO_4_ solution (Carl Roth Karlsruhe, Germany) to maintain stable 96% humidity.^19^, ^20^ Slides were placed inside the digestion chamber and incubated at 50 °C for 2 h. Afterward, four layers of 10 mg mL^–1^ CHCA matrix (Bruker) in 70% ACN (Honeywell), 1% TFA (Merck, Darmstadt, Germany) were applied using the TM‐Sprayer with parameters of 75 °C, 0.120 mL min^–1^ flow rate, and 1200 mm min^–1^ velocity.

### 2.3 MALDI–MS Imaging

MSI was performed in positive ion reflector mode over a mass range of *m/z* 600–3200 on rapifleX MALDI Tissuetyper TOF mass spectrometers (Bruker) fitted with a Smartbeam 3D Nd:YAG (355mm) laser. The Smartbeam parameter was set to M5 small, with the Imaging 50 µm application, 15 µm scan range, and resulting field size of 50 µm. Spectra were accumulated from 500 laser shots at 10 kHz frequency with a sampling rate of 1.25 GS s^–1^ and baseline subtraction performed during acquisition. The “Detector check” function was conducted regularly at each site in order to maintain this variable setting at appropriate voltages. For every measurement, the instruments were externally calibrated using the Peptide Calibration Standard II (Bruker),^12^ laser power adjusted according to on‐tissue test shots in order to reach the optimal ionization threshold, and two non‐tissue measurement regions included. Following MALDI–MSI, the matrix was eluted from the samples with 70% ethanol, stained with hematoxylin and eosin (H&E) and scanned with 20× objective magnification on a NanoZoomer SQ (Hamamatsu Photonics Deutschland, Munich, Germany), Aperio AT (Leica Biosystems, Buffalo Grove, IL, USA), or Mirax Desk slide scanner (Carl Zeiss MicroImaging, Göttingen, Germany). The H&E image was then co‐registered to the respective measurement in flexImaging v. 5.0 or SCiLS Lab MVS 2018b software (Bruker).

### 2.4 MS/MS Peptide Analysis

A rapifleX MALDI–MS/MS was used to confirm the identity of *m/z* 1105.6 directly from digested FFPE mouse intestine sections with TOF/TOF fragmentation. The generated spectrum was processed in flexAnalysis 4.0 (Bruker); the SNAP algorithm was used to pick monoisotopic peaks with S/N > 4, which were loaded into BioTools 3.2 (Bruker) and submitted to MASCOT (Matrix Science, Boston, MA). The MS/MS search was conducted against the SwissProt database for *Mus musculus*, with precursor and fragment ion tolerances of ±200 ppm and ±0.5 Da, respectively. Search criteria included variable modifications of protein N terminus acetylation, histidine/tryptophan, proline, and methionine oxidation, and up to three missed cleavages.

### 2.5 Data Analysis

MSI ion images were generated using flexImaging and SCiLS Lab with data normalized to the TIC.

#### 2.5.1 Scoring Spatial Resolution

Spatial resolution was scored by assessing the overlap of two mass signals characteristic of adjacent tissue regions in the mouse gut, designating one as “red” channel and the other as “green” channel. In R, zero intensities was set to the third percentile of the intensity values and maximum intensities to the 97th percentile. The dataset was then segmented: if the intensity of the less intensive channel in a pixel was at least 70% of the more intensive channel, the pixel was assigned to be a yellow pixel, otherwise the pixel would be assigned the color of the more intensive channel. Since this calculation would assign every pixel to be either “red,” “green,” or “yellow,” a spatial mask derived from the optical image was used to exclude pixels that were not measured on tissue. The spatial resolution score was calculated as the percentage of yellow pixels in the segmented image.

#### 2.5.2 Scoring Spectral Quality

Spectra from measured tissue regions were exported using the flexImaging “Export Spectra List” feature. A Windows Powershell script used to open 100 randomly selected spectra in flexAnalysis where a macro was used for peak picking on the selected spectra and collating a peaklist containing spectra number, *m/z* value, S/N ratio and intensity of each peak. The peak‐picking parameters were: “SNAP” algorithm, minimal S/N ratio 1, maximum number of peaks: 10 000. This peak list was further processed in R to calculate median number of peaks per spectrum, peaks above S/N 3, peaks over *m/z* 1500 and over *m/z* 2000. A 95% confidence interval for the median was calculated by using bootstrap‐resampling.

#### 2.5.3 Comparing Peak Intensities

For comparisons of peak intensities, the TIC normalized average spectra of each image was exported as CSV file to mMass and peak picking conducted with S/N 3.^21^, ^22^ Intensities for 17 different peptide *m/z* values from across the mass range and trypsin autolysis peaks were plotted with mean ± SD, and the co‐efficient of variance calculated (v.5.04, GraphPad Software, San Diego, CA).

#### 2.5.4 Spatial Segmentation

Spatial segmentation and PCA analysis were conducted in SCiLS. Datasets for comparison were combined into one file and preprocessed for a TOF instrument, with peak picking and alignment with weak denoising. The bisecting *k*‐means with correlation distance approach was used for spatial segmentation analysis.^23^ For the PCA, analysis was conducted on all individual spectra from the tissue regions, with weak denoising, and unit variance scaling.

## 3 Results

**Figure 1:**
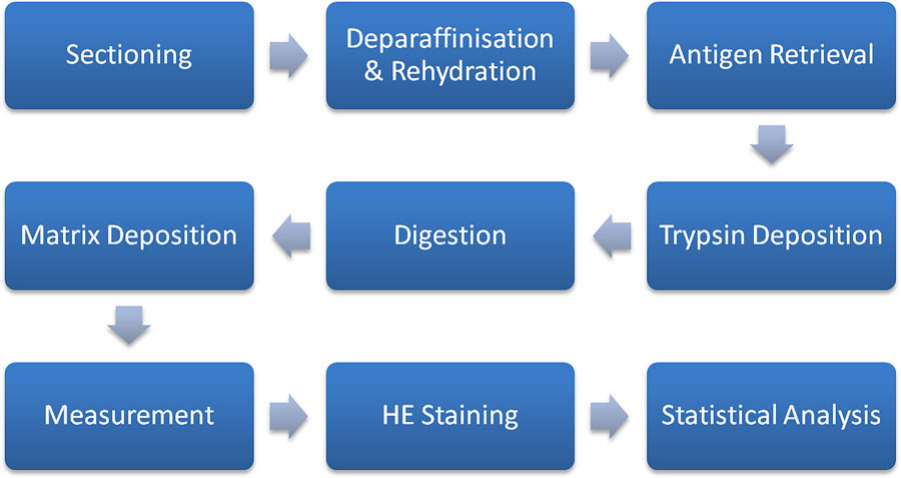
Schematic outlining the sample preparation workflow for peptide MSI of FFPE samples.

The aim of this study was to demonstrate the reliability and reproducibility of an improved in situ tryptic peptide digestion workflow developed for MALDI–MSI of FFPE tissues, which maintains spatial resolution. A schematic overview of the sample preparation is outlined in Figure 1, from which we identified "Trypsin Deposition", "Digestion," and "Matrix Deposition" as the most likely causes of spatial delocalization and preparation heterogeneity. To overcome this, our workflow uses an automated sprayer for enzyme application and matrix deposition. Saturated K_2_SO_4_ solution was used to maintain 97% humidity at 50 °C for the digestion. The efficacy of the protocol was tested on specially prepared animal tissue and clinical human samples.

### 3.1 Spatial Distribution

**Figure 2:**
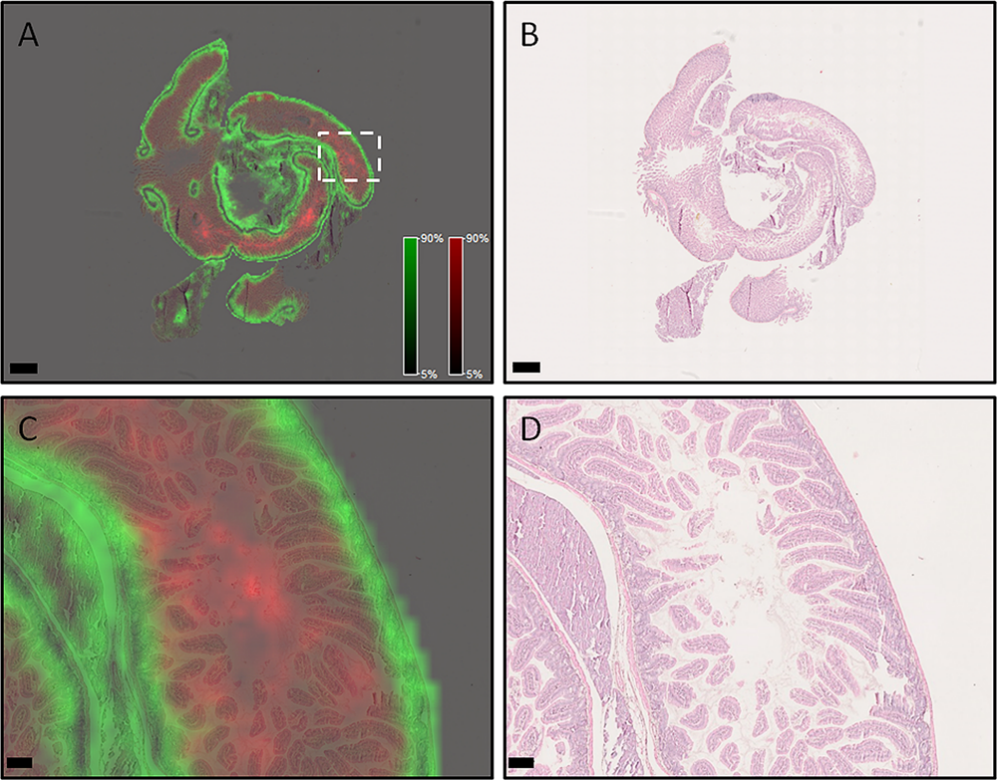
Spatial distributions of different *m/z* species are highly conserved in FFPE mouse intestine sample prepared with reported sample preparation protocol. A) Overview of mouse small intestine showing *m/z* 944.6 (red) in the villi and lumen, while *m/z* 1105.6 (green) is primarily in the crypts and muscle layers. Scale bar indicates 1 mm. B) Post‐measurement H&E stained sample. Scale bar indicates 1 mm. Higher magnification images (area denoted in [A] with white dotted box) of the *m/z* species (C) and H&E (D) overlay confirm the distributions. Scale bars indicate 100 µm.

Figure 2A shows the localization of two different *m/*z values in FFPE mouse intestine that has undergone MALDI–MSI measurement using our protocol. *m/z* 944.6 (red) and *m/z* 1105.6 (green) have distinctly different distributions in the mouse intestine. When viewed in the context of the post‐measurement H&E stained sample (Figure 2B), *m/z* 944.6 is primarily localized in the intestinal villi and lumen, while *m/z* 1105.6 corresponds to crypts and underlying muscular layers, which is confirmed at higher magnification (Figure 2C and D). MS/MS analysis identified *m/z* 1105.6 as a mouse collagen alpha 1(I) chain peptide (Figure S1, Supporting Information), fitting the distribution in muscle. In comparison, the distribution for *m/z* 1105.6 in sections digested with a wet tissue to maintain humidity is delocalized and exhibit ‘drop’ formations; likely due to condensation formed from uncontrolled humidity (Figure S2A and B, Supporting Information). To quantify spatial resolution, we used the Mixed‐Signal Approach that measures the overlap of two signals originating from adjacent tissues and calculates the proportion of yellow pixels in the entire image. The sample prepared with the new workflow had a spatial resolution score of 11.2% in comparison to 48.1% for the sample in Figure S2A, Supporting Information. The mixed pixel images of both samples are shown in Figure S2C, Supporting Information.

### 3.2 Application to Clinical Samples

**Figure 3:**
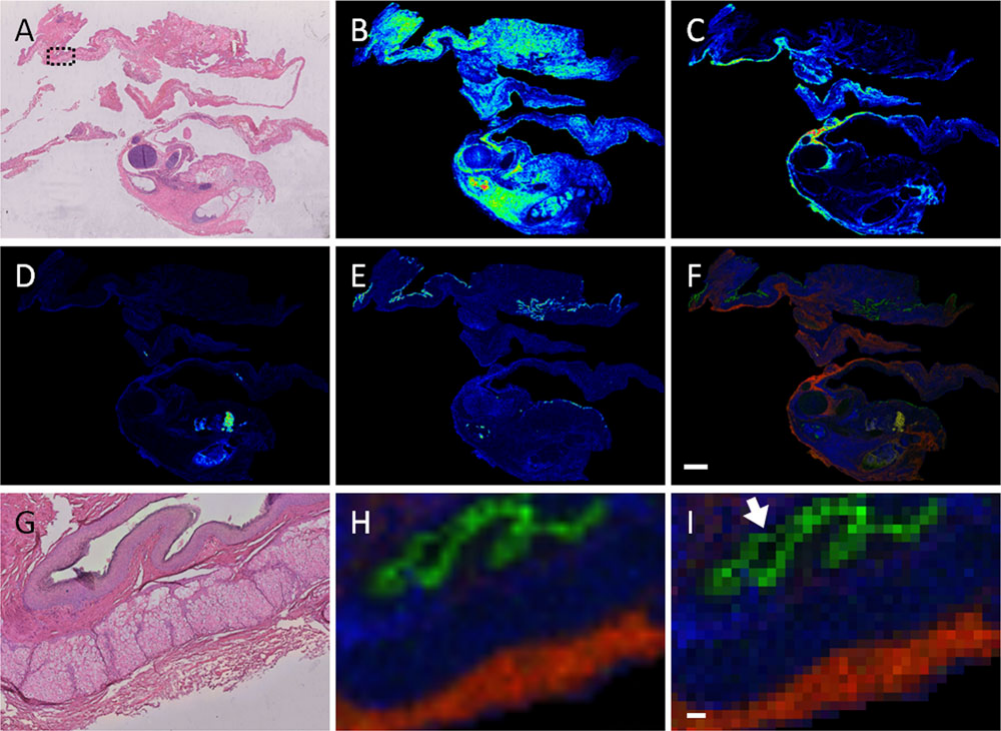
MALDI–MSI of human ovarian teratoma. A) Post‐measurement stained H&E human teratoma. B) MALDI–MSI image of *m/z* 1249.2 corresponding to smooth muscle and glands; (C; *m/z* 1095.7), connective tissue, (D; *m/z* 1127.7) mucus, and (E) *m/z* 1324.63 shows epidermal stratum corneum layer. F) Overlay image of the preceding ion images showing the individual localization of the ion images. Scale bar indicates 2 mm. G) Higher magnification of H&E stained sample; region indicated by dotted black box in panel (A). H) Overlay image of corresponding region from panel (G) shows the discrete localizations of the ion signals. I) Image of corresponding region with pixels representing individual MALDI spectra. In this view, it is possible to see that some ion signals are limited to a 50 µm region (arrow). Scale bar indicates 100 µm.

**Figure 4:**
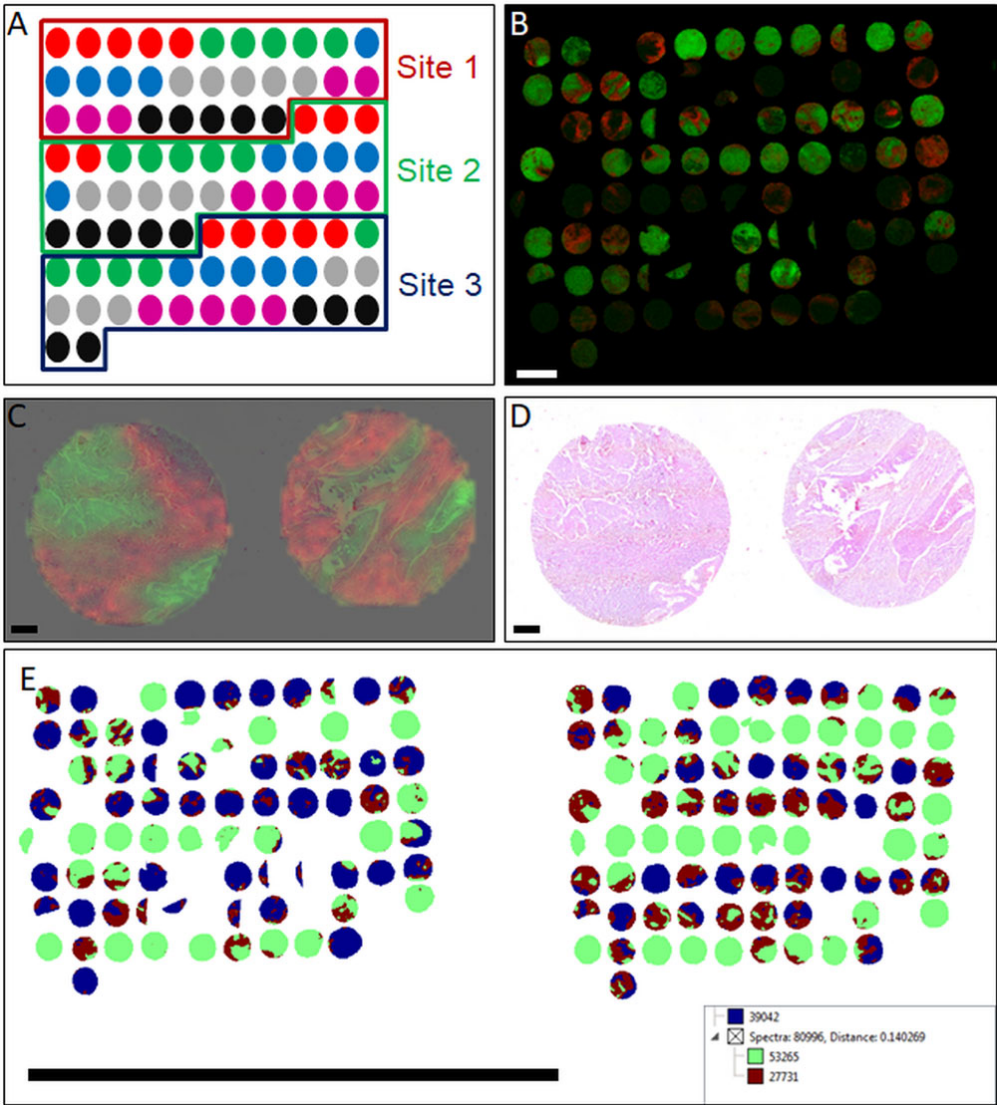
TMA composed of different tumors measured with MALDI–MSI. A) Layout of the multitumor TMA, showing the sample types and origin site. The samples and their color‐codes are: mantle cell lymphoma (red), seminoma (green), squamous cell carcinoma of the lung (blue), leiomyoma (grey), breast cancer (magenta), and melanoma (black). B) Ion images of *m/z* 788.6 (green) and *m/z* 1105.6 (red) show discrete localization on cores within the TMA. Scale bar indicates 2 mm. C and D) Higher magnification of SqCC cores ion images overlaid on the H&E indicate that *m/z* 788.5 (green) and *m/z* 1105.6 (red) localize to tumor and stroma, respectively. Magnified cores indicated in (A) with white dotted box. Scale bars indicate 200 µm. E) Segmentation analysis of two TMA measurements at different sites shows that the initial clusters (represented in blue, maroon, and green) does not separate in a way that reflects the measurement location or the sampling site. Scale bar indicates 3 cm.

To confirm if spatial resolution is also maintained in clinical samples, the protocol was tested on a human ovarian teratoma section (Figure 3) and a TMA composed of six different tumor entities collected from three different sites (Figure 4A). Co‐registration and independent assessment of the H&E stained section (Figure 3A) by a pathologist indicated that *m/z* 1249.2 corresponded to smooth muscle and glands (Figure 3B), *m/z* 1095.7 to connective tissue (Figure 3C), *m/z* 1127.7 to mucus (Figure 3D), and *m/z* 1324.63 to the stratum corneum layer of skin within the teratoma (Figure 3E). The discrete localizations of the ion signals are apparent when viewed at higher magnification (Figure 3G and H), while viewing the same image with pixels corresponding to individual MALDI spectra clearly demonstrate that some ion signals are limited to a 50 µm region. In the multitumor TMA, ion images show clear delineation of *m/z* 788.5 and *m/z* 1105.6 within the cores (Figure 4B). Higher magnification of SqCC cores in the TMA indicates that *m/z* 788.5 and *m/z* 1105.6 correspond to regions of either tumor or stroma, respectively, when overlaid on the co‐registered H&E (Figure 4C and D).

### 3.3 Reproducibility

Having confirmed the capability to produce highly spatially resolved data, we attempted to assess reproducibility of this workflow. Two sites conducted ten repetitions of the protocol on two different mouse intestine samples and data assessed for a variety of parameters. Using a novel QC workflow, no significant difference was found in the median number of peaks per spectrum, peaks above S/N 3, peaks over *m/z* 1500, or peaks over *m/z* 2000 (Figure S4, Supporting Information). Spatial resolution was also similar with means of 11.52 ± 2.91% and 10.85 ± 2.80% from sites 1 and 2, respectively. The intensities of 17 different peptide peaks across the mass range were also not found to be statistically significant (Figure S5, Supporting Information), while analysis of seven trypsin autolysis peaks indicates coefficients of variance ranging from 17.2% to 35.9% (Figure 6, Supporting Information).

**Figure 5:**
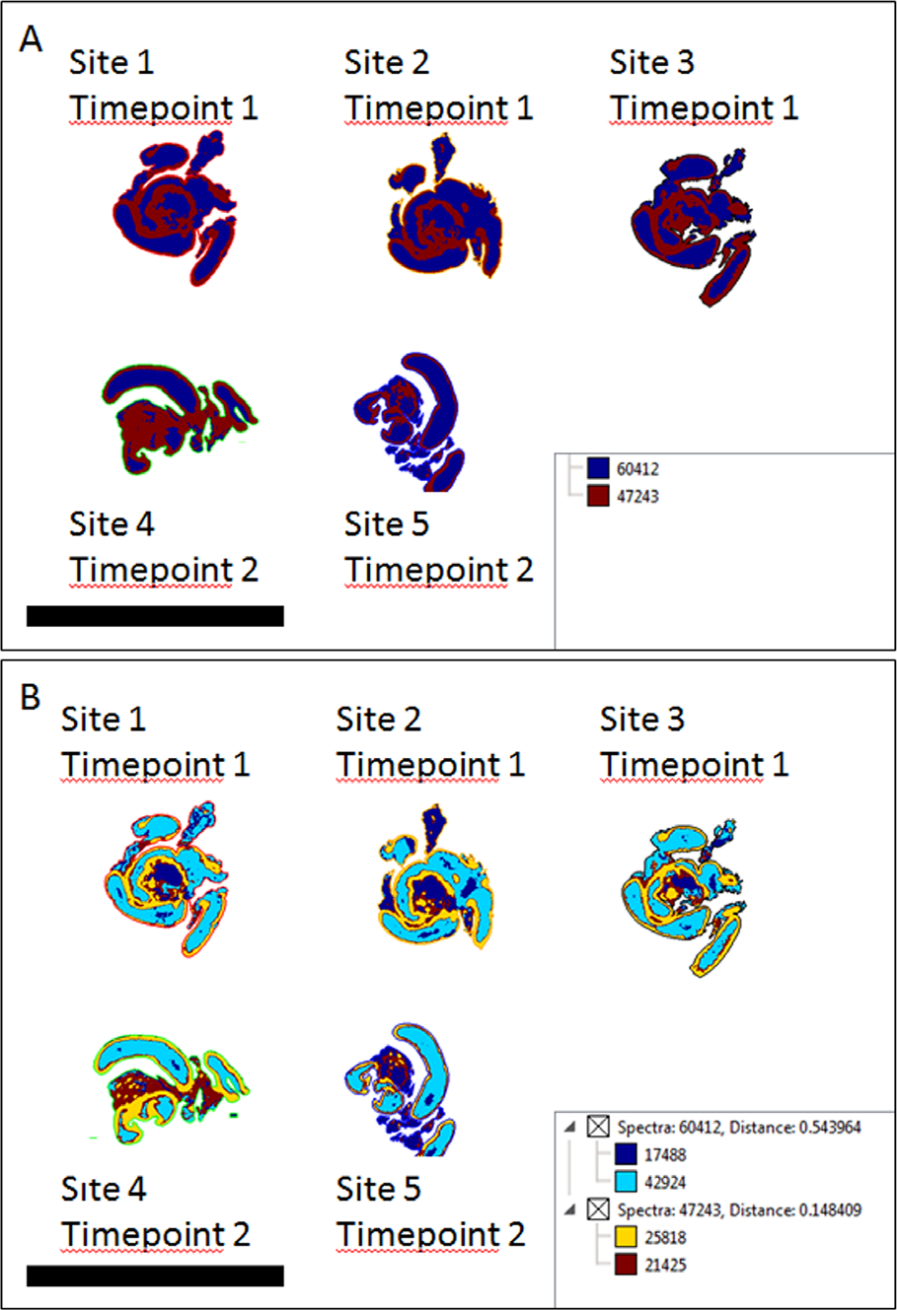
Segmentation analysis of mouse intestine samples measured as part of a multicenter study. A) The first level of clustering separates the spectra based on biological differences within the tissue (villi vs muscle—compared with Figure 2), rather than by location or time point. B) Higher levels of clustering continue along biological lines, also separating out non‐tissue regions. Scale bars represent 2 cm.

We conducted a multicenter study in which our standardized protocol was used at five different sites to prepare and measure mouse intestine sections. Three sites conducted the experiment on the same day, while two sites conducted theirs 1 month later. Analysis of the five datasets in SCiLS (Figure 5) indicates that the first few levels of segmentation are based on biology, between the villi and muscle layer similar to the result obtained in Figure 2, rather than on date or location of the preparation. A PCA of these five datasets (Figure S7A, Supporting Information; cluster images in Figure S7B, Supporting Information) shows that while individual spectra from site measurements cluster together, the spectra from different sites themselves do not form separate distinct clouds, indicating a lesser influence on data quality than the biology of the sample itself. Ion images of *m/z* 944.6 and *m/z* 1105.6 (Figure S7C and D, Supporting Information) show consistent localization in the lumen and muscular layer respectively across the sites, while for mixed pixel images the mean spatial resolution scores are consistent with similar data found in this study (Figure S7E and F, Supporting Information, compared to Figure S2C, Supporting Information).

To determine if original sampling location had the greatest influence in spectral quality, the multitumor TMA constructed from tissues originating from different sampling sites was measured at two sites and compared via spatial segmentation.^23^ Figure 4E shows that the highest level of segmentations divides the cores based on features within the cores, not along the sample origin nor the measurement date and location.

## 4 Discussion

MALDI–MSI is uniquely situated as a tool that allows the histologically defined mass spectrometric analysis of tissues. Although historically conducted on frozen samples, MALDI–MSI of peptides from FFPE tissues was first described a decade ago,^24^, ^25^, ^26^ and can be considered a technological breakthrough as the majority of tissue samples are preserved in this manner. Since then, a number of protocols for MALDI–MSI of peptides from FFPE samples have been published,^11^, ^12^, ^13^, ^27^, ^28^, ^29^, ^30^ indicating the importance of analyzing these samples to the preclinical and clinical community. Nevertheless, there are several limitations to the acceptance of MALDI–MSI as a clinical investigative tool. First and foremost, the preparation of FFPE samples for MALDI–MSI is perceived as being long and complex, requiring up to 2 days’ preparation.^12^ Second, the spatial resolution is inferior and the time required for MALDI–MSI analysis is greater when compared to traditional microscopy. Lastly, there are currently few multicenter studies demonstrating site‐to‐site reproducibility of MALDI–MSI using FFPE tissues or measurement of many samples collected from different sites.^16^, ^17^ In this study, we address these issues using an integrated workflow that results in highly spatially resolved data that is demonstrated to be reliably reproduced by different operators at a number of sites.

As with other MALDI–MSI protocols for FFPE tissues, our protocol contains deparaffinization and rehydration, antigen retrieval, trypsin deposition, and digestion steps prior to matrix application.^31^ Our workflow attempted to minimize the potential effect of instrument variability by using mass spectrometers and sample preparation devices of the same model and make from one manufacturer where possible. This extended to running the same spraying programs for enzyme and matrix application and setting guidelines to such as performing a regular detector check to maintain an appropriate voltage. As different sites in our multicenter study already had antigen retrieval devices, the established standard protocols for the respective devices were used, including different temperatures. Despite this, antigen retrieval does not appear to be the greatest source of variation in sample preparation. We also include post‐measurement H&E staining of the measured sample, which can then be co‐registered to the MS image during statistical analysis. While staining a consecutive section with H&E or IHC can also be conducted to assess molecular distributions,^28^, ^32^ the ability to directly stain the measured sample is more accurate due to the presence of section‐to‐section differences.^27^, ^33^

If spatial resolution is defined as the ability to distinguish structures within an image, several major aspects play a role in MALDI–MSI; the raster (laser diameter and spot‐to‐spot distance) used at the ‘Measurement’ step, the droplet sizes obtained during "Matrix Deposition" and "Trypsin Deposition", the homogeneity of the matrix coating, and analyte delocalization during the wet steps of the sample preparation. Although an early FFPE study utilized a 300 µm raster,^26^ improvements in laser technology have resulted in the development of instruments capable of 1 µm lateral step size.^34^, ^35^ Due to limitations from sample preparation, we used a 50 µm raster in an ultra‐high speed rapifleX MALDI Tissuetyper TOF instruments. This is not a major drawback as these instruments have acquisition rates up to 50 pixels per second due to the 10 kHz laser repetition rate.^36^, ^37^ With the described acquisition parameters, a tissue sample 4 mm in diameter can be measured in 6 min, or ≈130 min is required for the measurement of a 1.8 cm^2^ sized tissue section, both well within what is expected for a diagnostic time frame. We have used the overlap of mass signals characteristic for adjacent parts of the tissue to examine spatial resolution. The occurrence of “mixed color” pixels along tissue edges has been used qualitatively to assess spatial resolution before,^38^, ^39^ but visual appearance can be misleading and is not quantifiable.^40^ Our method quantifies this approach by calculating a spatial segmentation followed by counting the “mixed” pixels. It should be noted that even under perfect experimental conditions “mixed” pixels are expected, since with the 50 µm pixel size the tissue boundary cannot be fully resolved. Excess delocalization can be seen by an increase of “mixed” pixels. For tissues with comparable histology as the mouse intestine used here, this allowed us to compare the spatial resolution.

Matrix deposition is important as the crystals must be deposited in a reproducible, uniform manner with large enough wetness to extract analyte, but not so large as to lead to their delocalization. Likewise, trypsin application requires a balance between having a small droplet size but wet preparation—larger droplets produce better spectra but result in greater analyte delocalization.^27^ Our workflow uses the TM Sprayer, in which trypsin and matrix deposition is controlled by a series of parameters including nozzle temperature, nozzle speed, and matrix flow rate.^41^ Glycerol is included in the enzyme buffer to ensure sufficient moisture in the fine trypsin mist. Deposition of CHCA with the TM Sprayer has been compared to that of sublimated matrix,^42^ with crystal sizes as low was 1–3 µm reported for 2,5‐dihydroxyacetophenone matrix.^35^

For digestion, we implemented the principle of deliquescence—that a water‐soluble salt in its saturated solution will maintain a constant relative humidity in a closed environment.^19^, ^20^ Although a specialized instrument is commercially available,^27^ digestion is usually carried out in pipette boxes or Petri dishes inserted into an incubation oven with water or wetted paper to maintain humidity,^27^, ^30^ thus making it difficult to control parameters from site to site, or even from experiment to experiment. Additionally, such conditions can result in oversaturated conditions, causing condensation to form on the sample, leading to uneven digestion, as observed in Figure S2, Supporting Information. Using these aspects together in our protocol, the distribution of different *m/z* species was spatially conserved in all the tissues examined.

Having established parameters for maintaining spatial resolution, we examined if MALDI–MSI results could be obtained in a reproducible manner. Reproducibility can be defined in two ways: if different operators performing the same experiment at different sites can obtain similar results; and if similar data can be obtained from different tissue sources. Twenty repetitions of the same experiment conducted at two sites were found to produce a similar number of peaks of comparable intensity. It should be noted that while the results are not identical, due to inherent differences between tissue sections, it is not possible for users to have completely identical samples and cannot, therefore, reproduce identical results. Technical variation is also always expected to some degree even if identical samples would be measured under identical conditions. The possibility that MALDI tissue typing‐derived classifications will differentiate samples based on technical differences such as processing origin site rather than biological or pathological differences is one of the largest obstacles faced by MALDI–MSI. This is because the FFPE processing of tissues cannot be controlled; different processes exist and most of the critical information is missing, e.g., how long the sample stayed in the formalin. The spatial segmentation tool in SCiLS performs hierarchical clustering by dividing spectra into sets based on maximal difference.^23^ When measurements from a TMA constructed from samples collected at three locations and our multi‐center study were applied, the greatest discriminator was not sampling location or time and site of preparation but biology within the tissue. It demonstrates that reproducibility is possible when performed using our standard operating protocol.

## 5 Concluding Remarks

MALDI–MSI must be able to measure FFPE tissue for widespread applications. Using our standardized MALDI–MSI sample preparation protocol for the in situ tryptic digestion of FFPE tissues, it is possible to distinguish fine morphological structures in tissues reproducibly across different sites and time points. Additionally, we found that site‐to‐site variation in FFPE processing is not a major source of variation in MALDI–MSI analysis. These results support MALDI–MSI's potential to be an influential clinical tool.

## Conflict of Interest

A.L. and S.O.D. were employees of Bruker Daltonik GmbH for the duration of this study. The rapifleX MALDI Tissuetyper, HTX sprayer, and preparation protocol were provided by Bruker Daltonik GmbH as part of individual collaboration agreements between Bruker Daltonik GmbH, Proteopath GmbH, the Institute of Pathology, University of Heidelberg, and the Institute of Pathology, Technical University Munich..

## Supporting information

Supporting Information
