# Supplementary material for: Site‐to‐Site Reproducibility and Spatial Resolution in MALDI–MSI of Peptides from Formalin‐Fixed Paraffin‐Embedded Samples
Source: Proteomics Clin Appl. 2019 Jan 4;13(1):1800029. doi: 10.1002/prca.201800029 (PMC6590241; doi:10.1002/prca.201800029)
Supplement: Supplementary file 1 — Supporting Information [file PRCA-13-na-s001.pptx]

## Slide 1
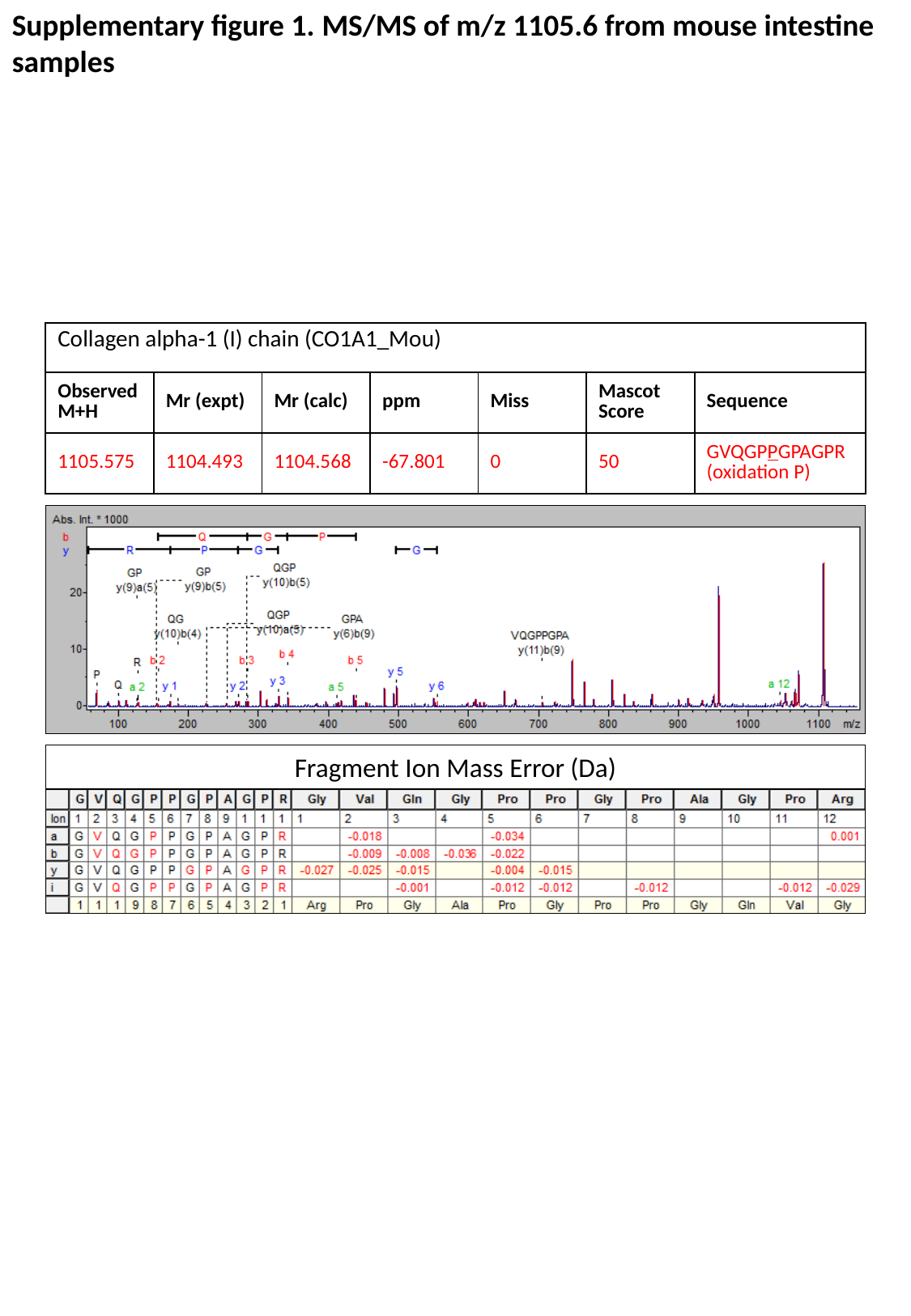

Supplementary figure 1. MS/MS of m/z 1105.6 from mouse intestine samples
| Collagen alpha-1 (I) chain (CO1A1\_Mou) | | | | | | |
| --- | --- | --- | --- | --- | --- | --- |
| Observed M+H | Mr (expt) | Mr (calc) | ppm | Miss | Mascot Score | Sequence |
| 1105.575 | 1104.493 | 1104.568 | -67.801 | 0 | 50 | GVQGPPGPAGPR (oxidation P) |
Fragment Ion Mass Error (Da)

## Slide 2
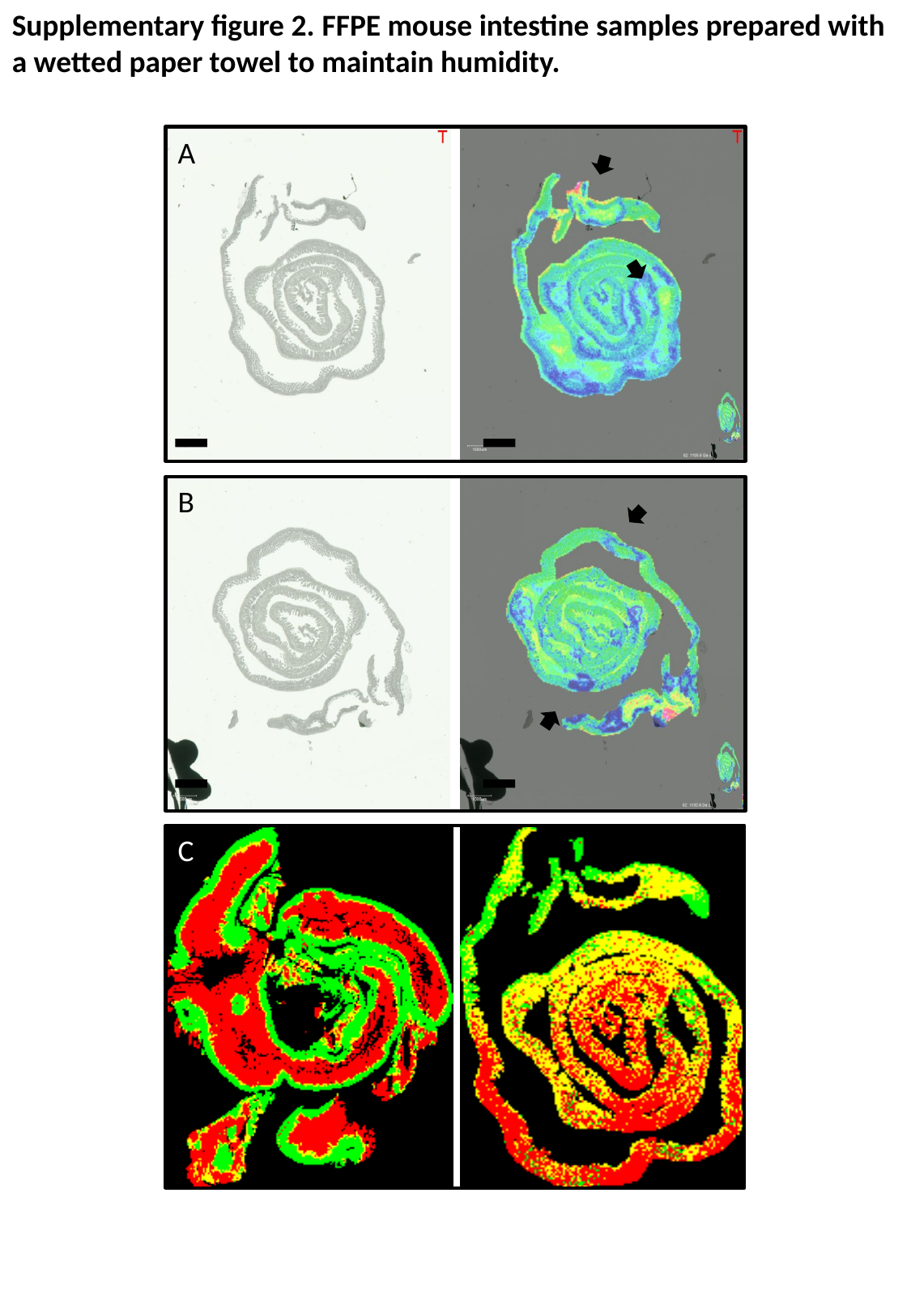

Supplementary figure 2. FFPE mouse intestine samples prepared with a wetted paper towel to maintain humidity.
A
B
C

## Slide 3
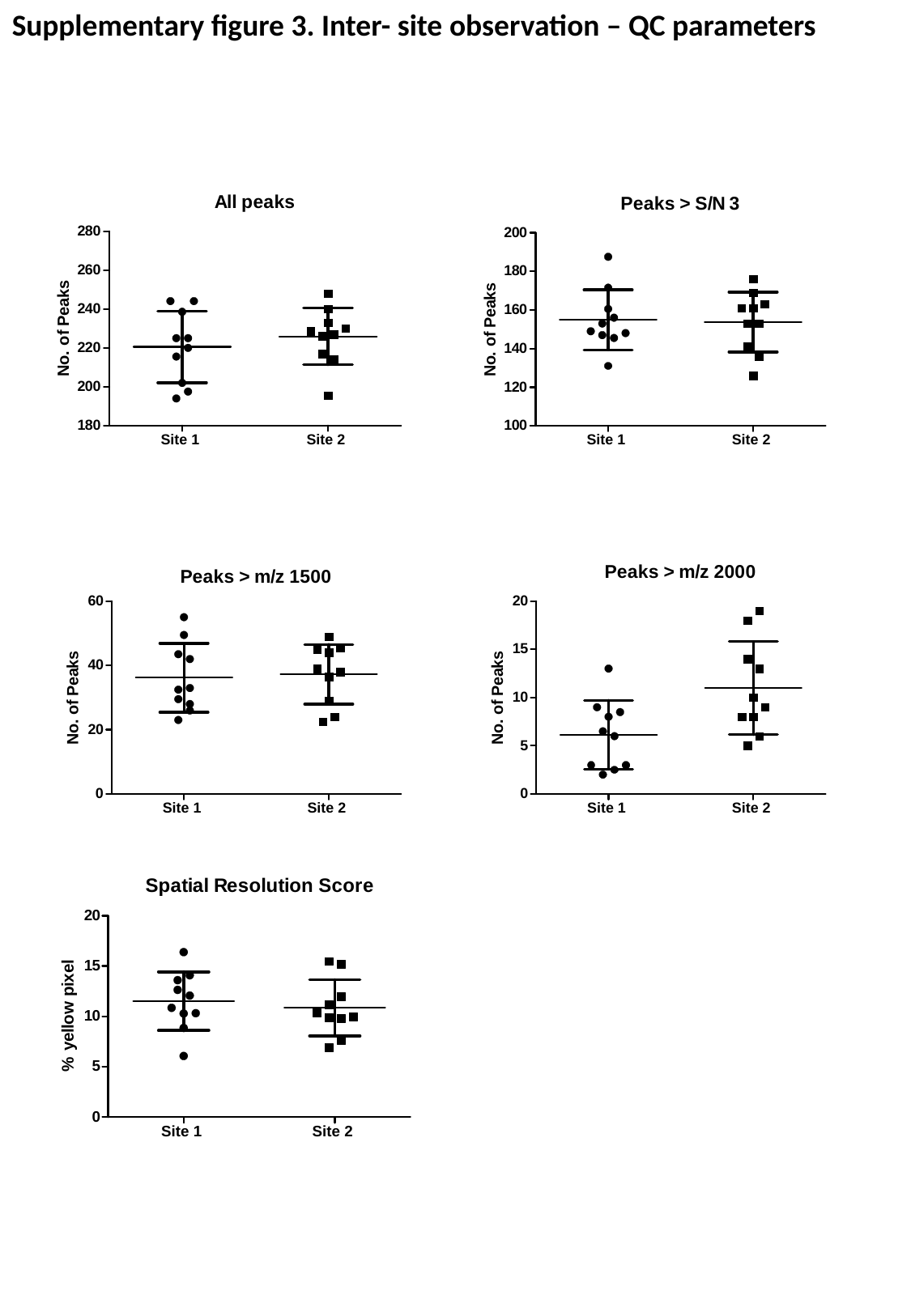

Supplementary figure 3. Inter- site observation – QC parameters

## Slide 4
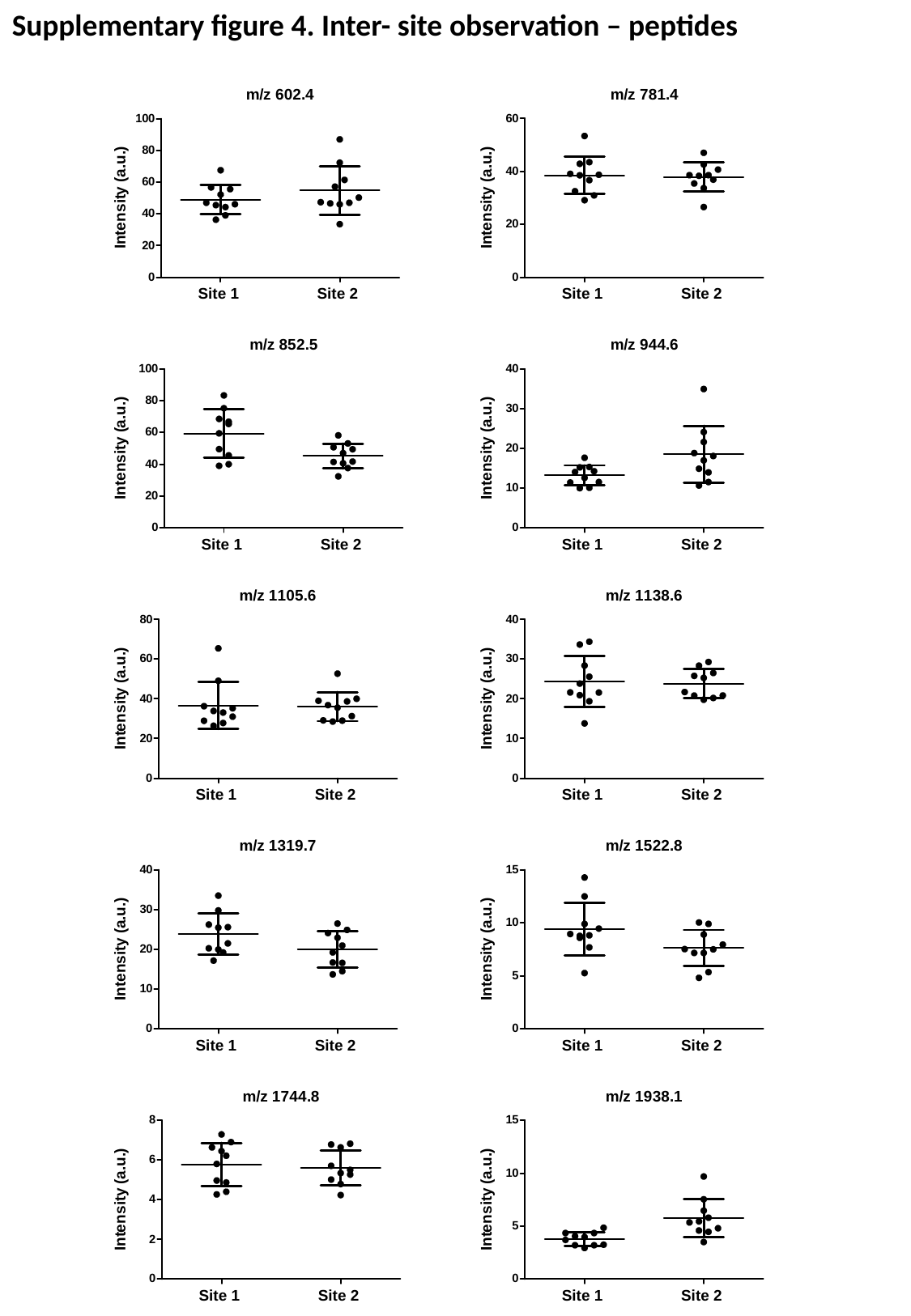

Supplementary figure 4. Inter- site observation – peptides

## Slide 5
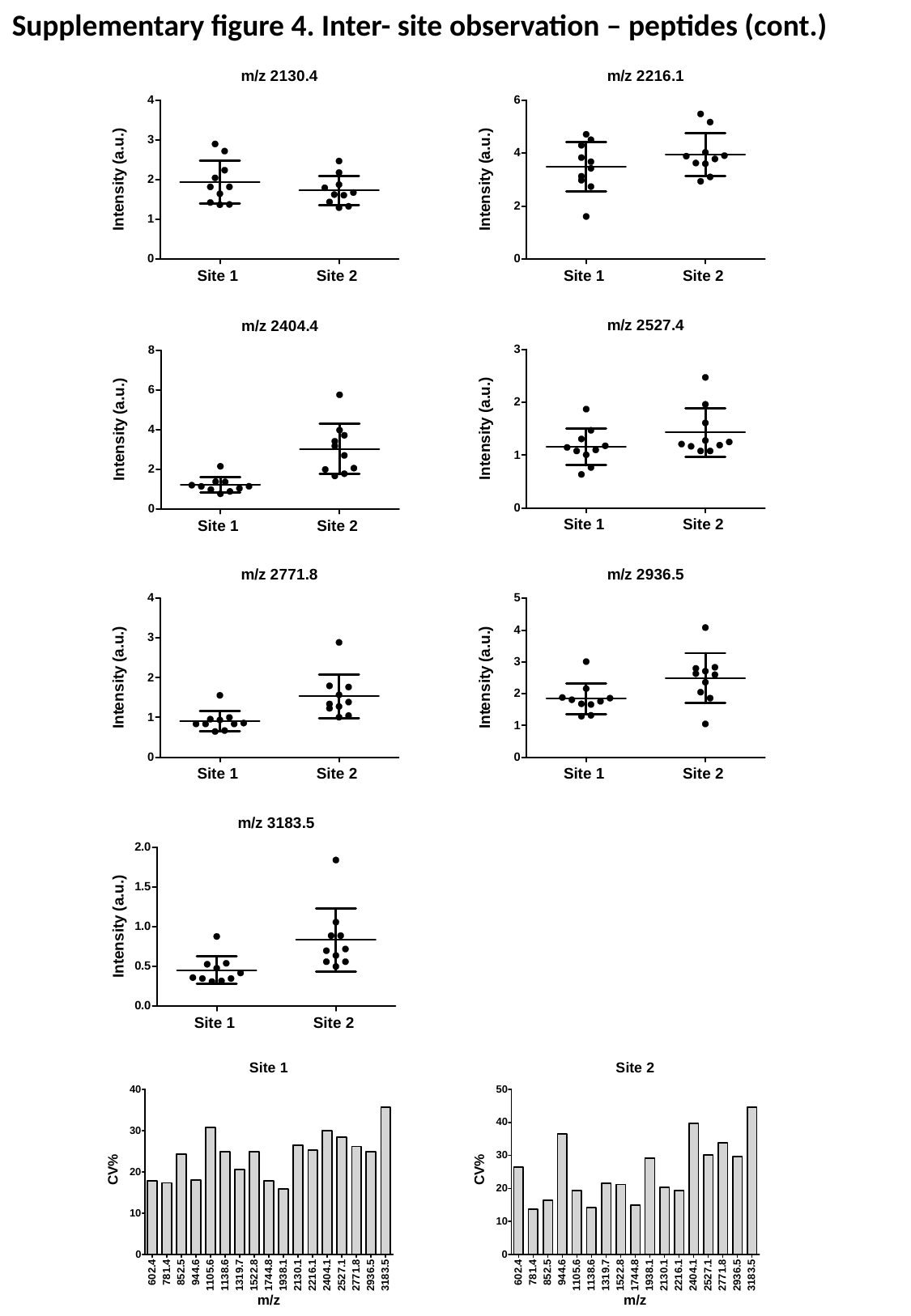

Supplementary figure 4. Inter- site observation – peptides (cont.)

## Slide 6
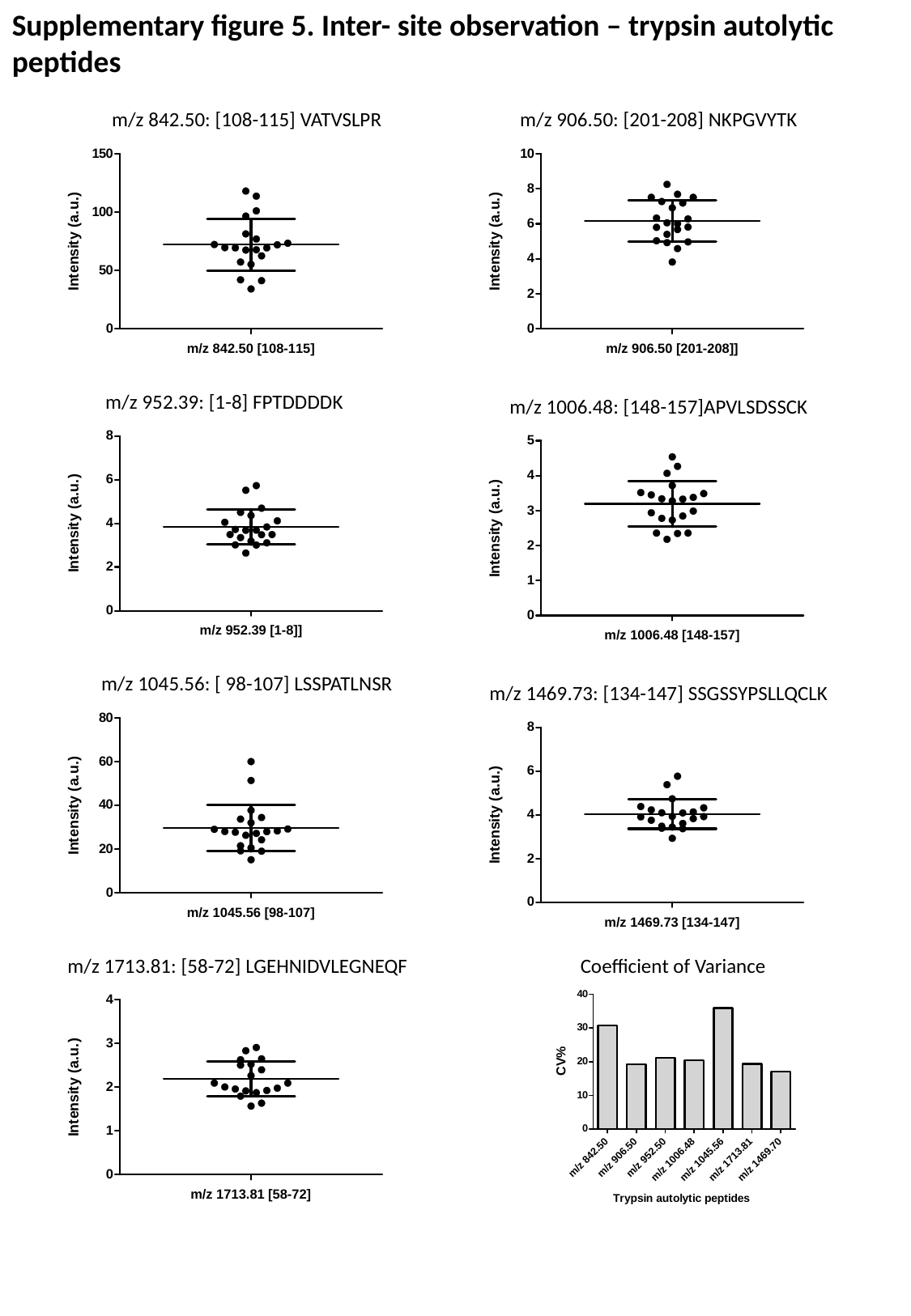

Supplementary figure 5. Inter- site observation – trypsin autolytic peptides
m/z 842.50: [108-115] VATVSLPR
m/z 906.50: [201-208] NKPGVYTK
m/z 952.39: [1-8] FPTDDDDK
m/z 1006.48: [148-157]APVLSDSSCK
m/z 1045.56: [ 98-107] LSSPATLNSR
m/z 1469.73: [134-147] SSGSSYPSLLQCLK
m/z 1713.81: [58-72] LGEHNIDVLEGNEQF
Coefficient of Variance

## Slide 7
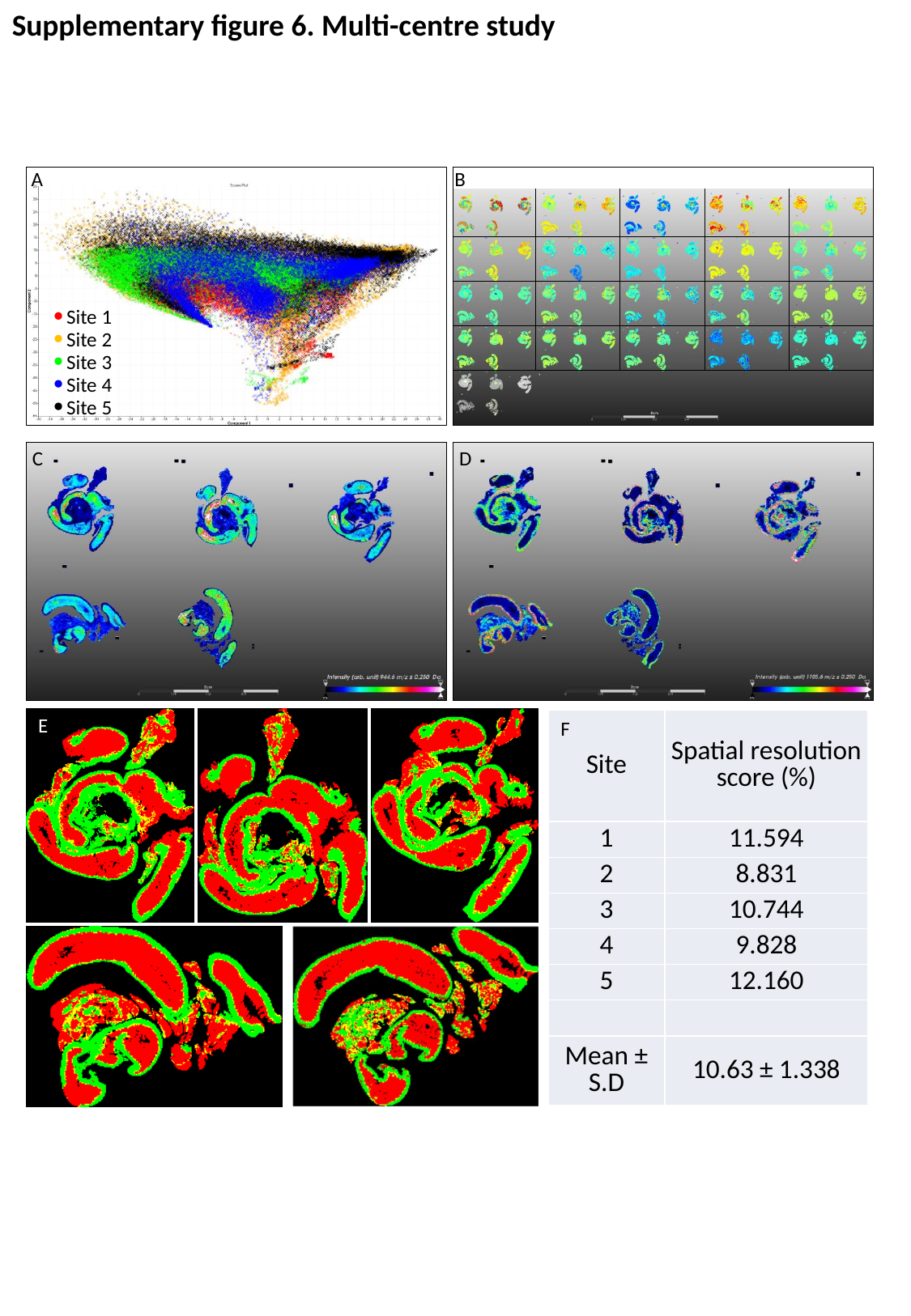

Supplementary figure 6. Multi-centre study
A
Site 1
Site 2
Site 3
Site 4
Site 5
B
C
D
E
| Site | Spatial resolution score (%) |
| --- | --- |
| 1 | 11.594 |
| 2 | 8.831 |
| 3 | 10.744 |
| 4 | 9.828 |
| 5 | 12.160 |
| | |
| Mean ± S.D | 10.63 ± 1.338 |
F
